# A Study of Landscape Features in the Coastal Area of the Seto Inland Sea Based on Landscape Paintings

**DOI:** 10.3390/ijerph20126165

**Published:** 2023-06-18

**Authors:** Yichuan Zhang, Shichen Zhao

**Affiliations:** 1 Graduate School of Human Environment Studies, Kyushu University, 744 Motooka Nishi-ku, Fukuoka 819-0395, Japan; zhang.yichuan.712@s.kyushu-u.ac.jp; 2 Faculty of Human Environment Studies, Kyushu University, 744 Motooka Nishi-ku, Fukuoka 819-0395, Japan

**Keywords:** landscape painting, landscape feature, similarity, cluster

## Abstract

Landscape paintings provide an abundant and objective representation of good and distinctive local scenery, which is widely used in local landscape analysis, so the comprehensive research of landscape paintings is fundamental and necessary for the subsequent landscape planning work. Landscape paintings include both planar information and spatial information. However, there has been little previous work on landscape paintings from both a three-dimensional and planar perspective, and the landscape features of landscape paintings have not yet been comprehensively clarified. Therefore, this paper, taking the Seto Inland Sea area as a case study, aims to comprehensively clarify the landscape features of the paintings and provide a valuable index of “good and characteristic landscapes” in this area based on the two planar features of element configuration and color, along with one spatial feature (element arrangement). To deeply clarify the typical landscape features of paintings, we attempt to propose a classification method by combining the similarity of features in different attributions. The results indicate that Sky, Green, and Sea are the most essential landscape elements, and yellow (orange), blue, and green hues are the most used in the paintings. In addition, the paintings were classified into eight typical landscapes, and seascape and field landscapes were the most significant presented in the landscape paintings in this area. This study presents a method to clarify the landscape features from both planar and spatial perspectives, providing more comprehensive guidance and data support for the subsequent landscape planning work and analysis—especially in regional landscape exploration—and for the development of tourism landscape resources in urban planning.

## 1. Introduction

With economic growth, landscape problems such as homogenization, destruction, and disharmony have emerged and become an international issue. Thus, many countries have successively published relevant laws and regulations. Japan passed the Landscape Law in 2004 to protect the local landscape [1]. Since then, local governments and organizations have begun to develop their own landscape planning, an essential part of which is the preservation and regeneration of “good scenery” mentioned in the Landscape Law [1]. At the same time, in addition to conducting questionnaires and expert evaluations, scholars have also used local resources to explore excellent local landscape characteristics from an academic perspective, such as local landscape paintings [2,3,4,5,6,7,8], poems [9], folk songs [10], etc. Among them, landscape paintings provide a more abundant and objective representation of the “good and distinctive local scenery” mentioned in the Landscape Law, and so they are widely used in local landscape analysis; thus, comprehensive research on landscape paintings is fundamental and necessary for the subsequent landscape planning work. 

Landscape paintings include both planar information and spatial information. Planar information takes the landscape painting as a two-dimensional image, focusing on composition, configuration, color, texture, brushwork, artistic style, etc. Spatial information takes a three-dimensional perspective, focusing on the relationship between the observer (painter) and the viewed scenery, including visible and invisible objects, distance, angle, depth, depression angle, elevation angle, etc. [11]. Previous landscape analysis of landscape paintings has mainly focused on the two aspects of landscape: elements and viewpoint area. In terms of landscape element analysis, for example, Hagishima [2] clarified the significant landscape elements into different spatial distances (i.e., short, medium, and long) and classified the landscapes into six groups based on 19th-century European oil landscape paintings. Shimizu [5] and Iwasaki [6] deeply studied one essential landscape element depicted in landscape paintings. In terms of viewpoint analysis, Ogawa [7] proposed a method of estimating the viewpoint from the information of urban landscape paintings and urban space using camera calibration and 3CG techniques. Sakai [8] proposed a search method for viewpoint exploration based on the significant indicators of landscape elements and viewpoints with the help of mesh data. These previous studies have developed many methods and drawn numerous academic findings with respect to landscape clarification and viewpoint exploration based on landscape paintings; however, most of them studied the characteristics of landscape paintings from a spatial perspective, while fewer considered the planar features, leading to a partial loss of information on landscape features. In the image analysis field, the research on landscape paintings has mainly focused on the semantic segmentation model [12], artistic style analysis or classification [13,14,15], color [16], and composition [17] analysis related to landscapes. Choi [16] extracted color code information from Monet’s landscape paintings and generated a palette with biological characteristics, providing a comfortable color palette reference for interior architecture with natural landscapes. Lee [17] researched the proportional compositional features of landscape paintings in different styles and periods through information entropy theory. However, analyses of landscape element features and spatial features of landscape painting are relatively lacking in image work. Through these previous works, landscape analysis of landscape paintings has been discussed from spatial and planar perspectives; however, little work has examined landscape paintings from both three-dimensional and planar perspectives, and the landscape features of landscape paintings have not yet been comprehensively clarified.

In planar information, an image contains so much information that simply analyzing the entire image would inevitably lead to significant bias in landscape analysis. To avoid interference from other information in landscape feature analysis, the features that are highly related to the landscape’s element configuration and color features can be selected. In terms of spatial information, landscape elements at different distances have commonly been analyzed in previous studies [2,3,4,5,6,7,8], which can simplify and effectively clarify the spatial arrangement features of landscape elements; other features, such as angle or depth, put more emphasis on viewpoint features, which require more information about the viewpoint where the painter painted. Thus, in this study, we selected three features to comprehensively analyze landscape paintings: (1) the element configuration, (2) the color as planar information, and (3) the element arrangement as spatial information. In this paper, the element configuration feature refers to the distribution, type, and proportion of elements in the image [18]. The color feature refers to the global color features of the landscape painting, which can reflect the color features of the significant elements or the scenery in different time periods to a certain extent (such as dusk, sunrise, night, etc.). The element arrangement feature refers to the arrangement of landscape elements in the short-distance view, medium-distance view, and long-distance view [11]. 

To deeply clarify the landscape feature, cluster analysis is widely used to further exploration. The main step of clustering is to count the values of samples in various indicators, calculate the distance between samples employing the values and distance metric method, and perform clustering based on the distance [19]. However, in this study, there were certain differences in analysis methods between spatial features and image features. Specific indicators clarified the element arrangement features, while the element configuration features were clarified according to the multiple pixels in the image, which is hard to extract the specific indicators. Therefore, in this paper, we attempt to calculate the similarity (dissimilarity) between samples from three aspects separately and add these three similarities (dissimilarities) to obtain the overall similarity as the distance for cluster analysis. As mentioned above, the historic landscape of the Seto Inland Sea region was gradually destroyed as the economy developed and was engulfed in resort and industrial development. Seto Inland Sea area has not only been recognized as a place with Japan’s most representative island-sea-urban scenery, but has also been considered a world-renowned holy land of art. Many painters were born here and left behind many realistic landscape paintings. In previous works on the landscape of the Seto Inland Sea, Nishida [20] analyzed the changes of preferences in sea view based on the ancient literature, Iwamoto [21] clarified the general landscape features based on the landscape painting in Chugoku region, and Zhang [22] further clarified landscape characteristics and proposed the utilization method for the viewpoint exploration. However, they all studied the spatial landscape characteristics, as mentioned before. Therefore, taking the Seto Inland Sea coastal oil painting as an example, we aim to comprehensively study the landscape features of the painting from three aspects: element configuration, color, and element arrangement, and propose a landscape classification method based on the similarity between samples to classify landscape features in the coastal area of the Seto Inland sea, to provide a valuable index of “good and characteristic landscapes” from both image perspective and spatial perspective in this area.

## 2. Materials and Methods

Firstly, based on landscape paintings, we studied similarity matrices and general features of element configuration, color, and element arrangement. Secondly, the three similarity matrices would be used to further cluster analysis. 

### 2.1. Seto Inland Sea Area and Landscape Paintings 

**Figure 1:**
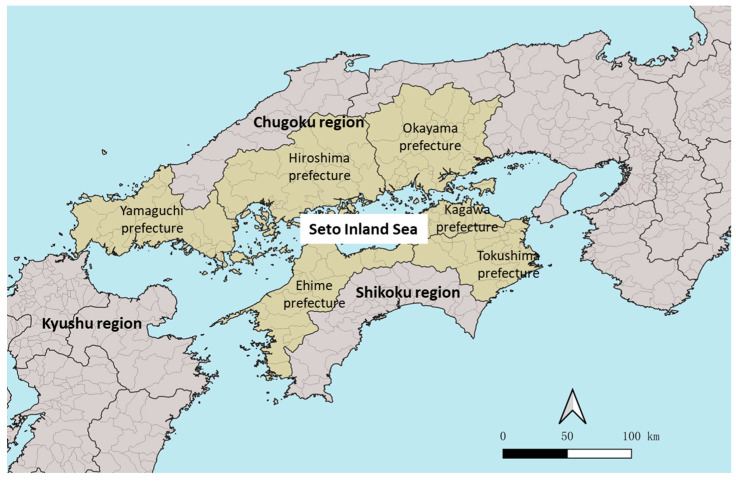
Study area in the coastal area of the Seto Inland Sea in Japan.

The Seto Inland Sea is an inland sea surrounded by the Honshu, Shikoku, and Kyushu regions. Particularly, Chugoku and Shikoku regions are connected by three major series of bridges, and there are many famous sightseeing spots and modern art destinations around these two regions of the Seto Inland Sea [22]. Therefore, in this study, we focus on the coastal area of the Seto Inland Sea located in the Chugoku and Shikoku regions, including Yamaguchi, Hiroshima, Okayama, Ehime, Tokushima, and Kagawa prefectures. The study area in the coastal area of the Seto Inland Sea is shown in Figure 1.

Realistic oil paintings or woodcuts are usually utilized for the study to ensure the authenticity and distinguishability of the scenery and elements depicted in the painting. This study selected the sample landscape paintings from 90 paintings used in Zhang’s research [22]. These 90 landscape paintings in Zhang’s research were collected from prefectural museum art collections and art books. To reduce errors due to the various sizes of painting, we calculated the aspect ratio of each painting and chose a ratio of 4:3 which is close to the size of 62 of 90 paintings. Therefore, these 62 paintings were selected as research samples for this study and were normalized to the size of 960 × 720 pixels. The detailed information is shown in Appendix A.

### 2.2. Element Configuration Features 

From Zhang’s research [22], eleven main landscape elements depicted in landscape paintings of the Seto Inland Sea area were selected for element configuration analysis, including Green (tree, forest, grass, and field), Sky, River/Pond, Boat, Mountain, Island, Sea, Mountain/Island, Open Space, Architecture (building and bridge), and Others.

**Figure 2:**
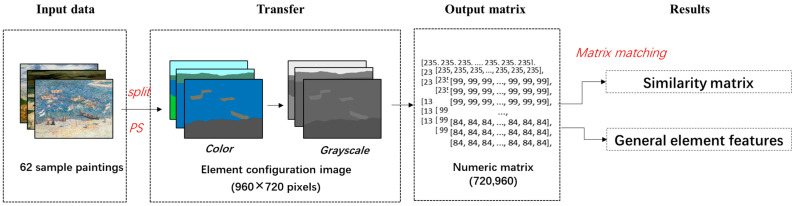
Research flow chart of element configuration features.

In this section, we first split the landscape elements with Photoshop software to generate an element configuration image. Then we transferred the image to grayscale to output the grayscale value matrix (hereinafter referred to as “matrix”). The 62 matrices were used to analyze the configuration similarity and general features among sample paintings. The flow chart is shown in Figure 2.

The matrix contains element configuration information, including the distribution of element position (aij), element type (Pixel value), and proportion (Number of values). Therefore, the similarity of element configuration can be considered as the matching degree of pixel values in the matrix. Considering that distance metric is computed by pixel values, which means that different color assignments to elements will lead to different results, we applied proportion metric to measure the matrix degree, i.e., element configuration similarity, calculated the proportion of the number of pixels with the same value at the exact location between two matrices—that is the proportion of the 0 values obtained by subtracting two matrices. The pixel value is meaningless and can be labeled to any value if a different element corresponds to different pixel values.

It should be noted that when generating the element configuration image by splitting the elements from the landscape painting with the help of Photoshop software, the resolution of the selection would lead to averaging of pixels with domain pixels at the boundaries between elements, which means that the output pixel matrix will have error values which outside the eleven values corresponding to these eleven elements (or incorrectly averaged to the values corresponding to other elements). The average number of error values accounts for approximately 0.015% of the total, and it will not significantly impact the similarity results.

### 2.3. Color Features

**Figure 3:**
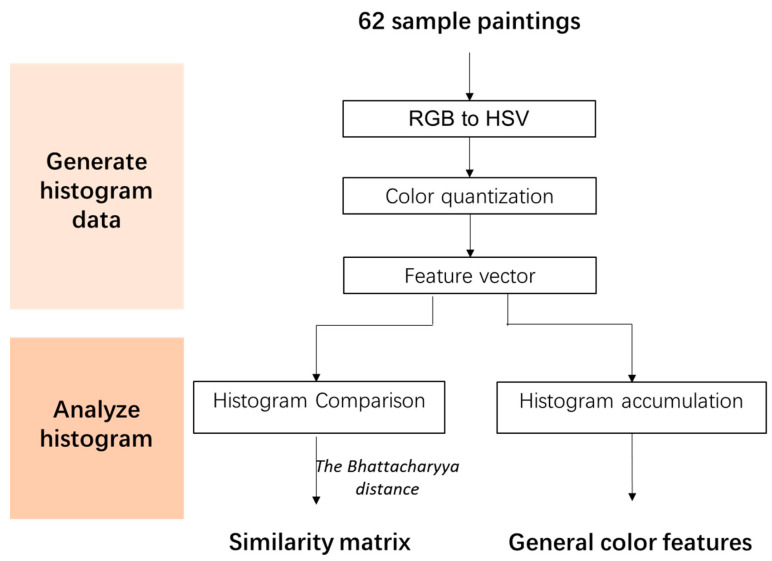
Research flow chart of color features.

In this section, we extracted the color features of paintings and utilized color histograms to help visualize the color features. This section can be divided into two steps. Firstly, we generated the histogram data based on the quantization scheme. Secondly, we clarified the general color features and calculated the similarity among paintings based on the histogram data. The research flow chart shows as follows (Figure 3):

#### 2.3.1. Histogram Data

RGB color space and HSV color space are most used for presenting colors. RGB color space represents the color through three channels, including red (R), green (G), and blue (B) [23]. The different combinations of these three channels can form almost all colors that human vision can perceive, such as the yellow color in RGB (255, 255, 0) and the white color in RGB (255, 255, 255). However, the color is determined by three channels, leading to excessive dimension and weak visualization, while grayscale (one channel) would lose many color features. For these reasons, we used the color model of HSV (Hue, Saturation, and Value) color space to analyze color features. Hue is measured by angle, with a value range of 0 to 360°, representing color information, and each color is not uniform according to the hue range [24]. Saturation represents the concentration of the color, and Value refers to the brightness of the color, which are both measured by percentage, from 0% to 100% (or 0 to 1) [24].

**Figure 4:**
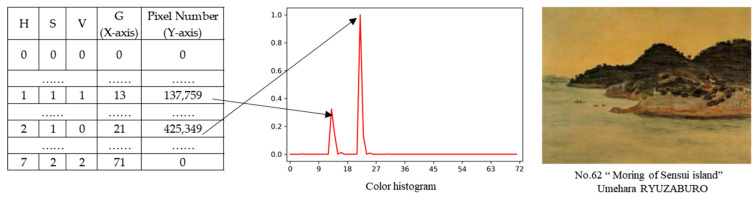
Color histogram of sample No.62.

Color histogram, which can reflect the composition and proportion of colors in the image, is widely used to illustrate the color features [25]. However, there are usually too many colors contained in a painting, resulting in a high dimension of the histogram vector. Therefore, quantizing the HSV space into several bins is necessary to reduce the dimension and can also weaken the impact of color aberration caused by printing to a certain degree. Here we used the quantization scheme proposed in Li’s research [26]. Li divided the Hue space into eight parts, Saturation space, and Value space into three parts, and constructed function G to calculate the sum value of H, S, and V. We calculated the color histogram of 72 bins (8 × 3 × 3 = 72) based on the quantization scheme and function G. The specific quantization scheme and one example are shown as follows. (Table 1, Figure 4).

#### 2.3.2. Histogram Similarity 

This paper applied the Bhattacharyya distance, which is widely used to measure discrete probability distributions, to compute the color histogram similarities among paintings. The calculation formula for the Bhattacharyya distance is as follows:

(1) $$d\left(H_{1},H_{2}\right)=\sqrt{1-\frac{1}{\sqrt{{\bar{H}}_{1}{\bar{H}}_{2}N^{2}}}\underset{I}{\sum }\sqrt{H_{1}\left(I\right)\cdot H_{2}\left(I\right)}}$$

where N is the number of histogram bins. In this paper, N = 72. $H_{1}$ refers to the histogram vector of sample 1. ${\bar{H}}_{1\;}$ refers to the average value of the histogram vector of sample 1. $H_{1}\left(I\right)$ is the value of the pixel number of sample 1 in the *I*-th bin.

The value of the Bhattacharyya distance is distributed in [0, 1], where the value closer to 1 indicates greater similarity, while the value closer to 0 suggests more difference in color features between the two paintings. 

### 2.4. Element Arrangement Features

Zhang [23] selected nineteen landscape categories and three distances (short distance, medium distance, and long distance), and identified the distance type of each category with binary data (0 indicates absence, 1 indicates presence). The binary database was used for the following analysis in this paper. We applied the Jaccard index method, which is commonly used in binary data to calculate the distance, for the similarity analysis of arrangement. The Jaccard coefficient is defined as the ratio of the size of the intersection of *A* and *B* to the size of the union of *A* and *B*. The calculation formula is as follows.

(2) $$J\left(A,B\right)=\frac{\left|A\cap B\right|}{\left|A\cup B\right|}$$

The value of the Jaccard coefficient ranges from 0 to 1, where the value closer to 1 indicates greater similarity, whereas the value closer to 0 suggests distinct of arrange features between the two paintings. 

### 2.5. Comprehensive Similarity Features and Classification

A comprehensive similarity matrix was obtained by adding these three similarity matrices. The similarity matrix would be transferred into the distance (Dissimilarity) matrix to conduct a cluster analysis through Hierarchical Clustering Analysis. The distance value in the comprehensive distance matrix was distributed in [0, 3]. 

## 3. Results

### 3.1. Element Configuration Features

**Figure 5:**
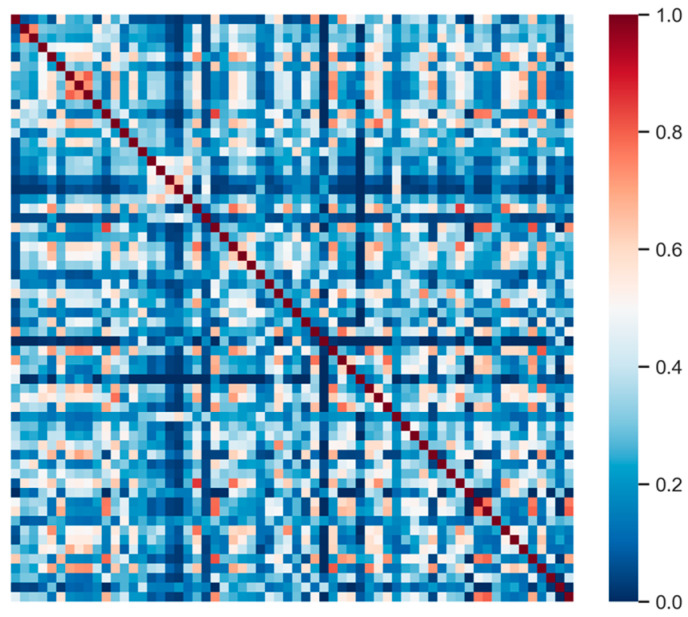
Similarity matrix of element configuration among 62 paintings.

Through the matrix pairwise comparison, the similarity matrix of element configuration is obtained and illustrated as a heat map in Figure 5.

**Figure 6:**
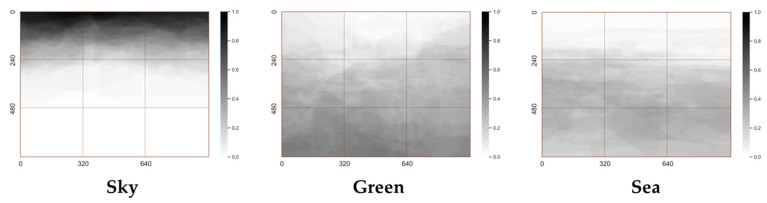
The distribution and probability of the elements.

As for the general element configuration feature, we calculated these aspects of each element: (1) the number of the painting which depicted it; and (2) the average percentage in the entire painting. The distribution and probability are depicted in the painting image (Figure 6). The results are interpreted as follows: 

Sky, Green, and Sea were depicted in more than half of these paintings, with 57, 42, and 36 paintings depicting them, respectively. In addition, Sky, Green, and Sea elements account for about 68% of the entire image, with Architecture and Mountain accounting for 9%, respectively. The Sky element was almost always depicted at the top part of the image. By comparison, the Green and Sea elements were less depicted and were more evenly distributed in the image, of which the Sea element was more inclined to be depicted in the middle and bottom parts of the painting. In general, Sky, Green, and Sea were the most essential elements of the painting of the Seto Inland Sea area.

### 3.2. Color Features

**Figure 7:**
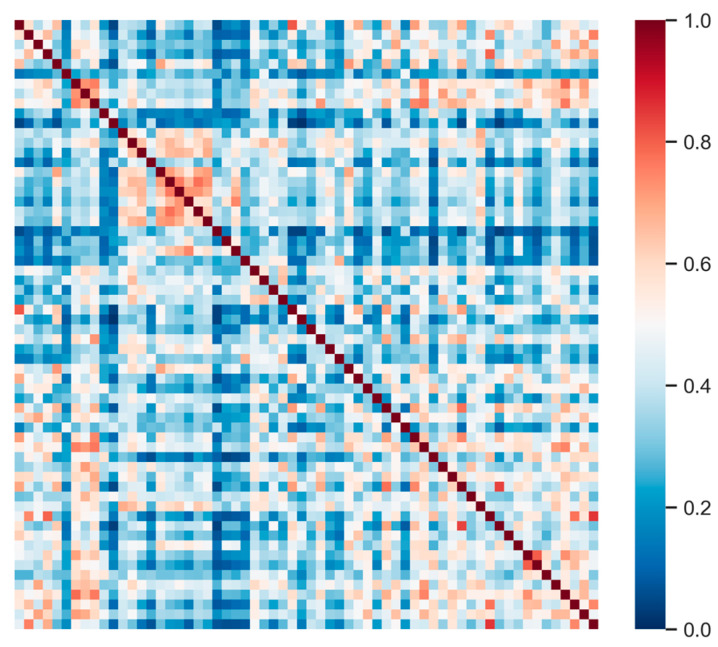
Similarity matrix of the color feature of 62 paintings.

Through the color histogram pairwise comparison, the similarity matrix of color features was obtained and illustrated as a heat map in Figure 7.

**Figure 8:**
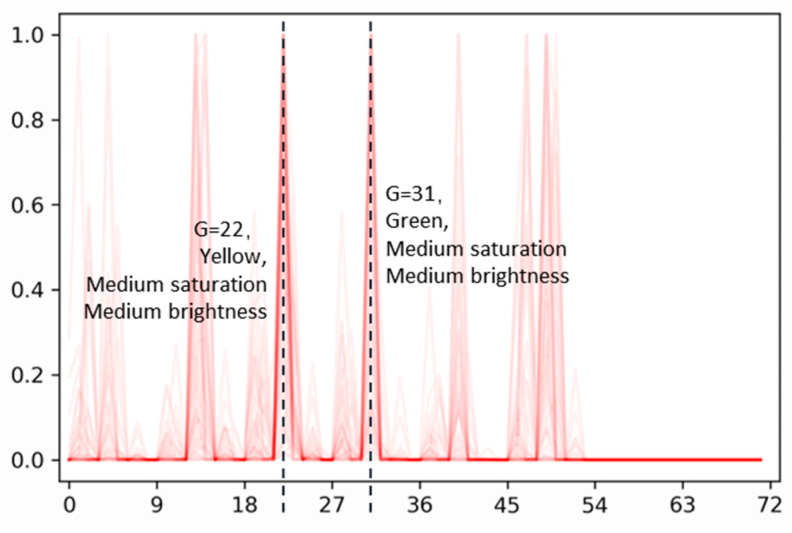
Color histogram of 62 paintings.

**Scheme 1:**
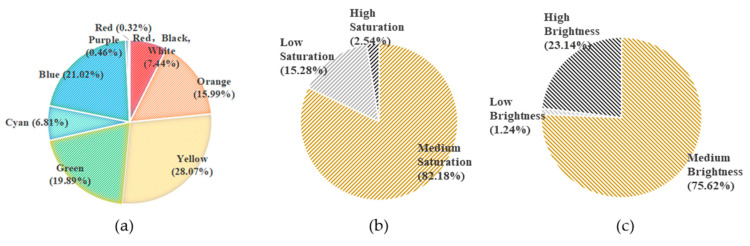
Hue, Saturation, and Brightness features of 62 paintings. (**a**) Hue feature, (**b**) Saturation feature, and (**c**) Brightness feature.

A histogram and diagram were applied to help visualize the general color feature. We adjusted the transparency of each painting to about 0.016 (1/62). We overlayed all 62 histograms to one histogram, shown in Figure 8, in each bin, where the density of the line color represents the appearance frequency. The line height represents the number of pixels occupied in the entire image. The darker and higher it appears, the more paintings are colored, whereas the lighter and shorter it appears, the less color is used. Since the weight of H (hue) is much higher than that of S (saturation) and V (value) (G = 9H + 3S + V), it is feasible to determine the approximate hue from the color histogram, such as a yellow or blue hue. However, it cannot intuitively reflect the color’s saturation and brightness, so we further measured the saturation and brightness feature and presented them in Scheme 1.

From the histogram in Figure 8 and Scheme 1a, yellow (28.07%), blue (21.02%), green (19.89%), and orange (15.99%) were mostly used in target paintings, and red and cyan followed by 7.44% and 6.80%. As for the saturation shown in Scheme 1b, medium saturation was dominant, accounting for 82.18% of the total pixels, and low saturation was followed by 15.28%. In terms of brightness shown in Scheme 1c, medium brightness was also largely used in target images accounting for 75.62%, and high brightness followed with 23.14%. In addition, as the dotted line shows in Figure 8, the G value of 22 (refer to a yellow hue, medium saturation, and medium brightness) and 31 (refer to a green hue, medium saturation, and medium brightness) were mostly painted, which accounts for 19.89% and 21.02%, respectively.

From the results, we can briefly summarize- that yellow, blue, green, and orange were commonly used in target paintings, red and cyan were also presented in some paintings, while purple was rarely used. In addition, paintings were usually painted in medium and low saturation, as well as medium and high brightness. The yellow and green with medium saturation and brightness were mostly presented in the landscape paintings of the coastal area of the Seto Inland Sea. The significance of the green and blue hue was consistent with the element features clarified in 3.1, for these three elements were usually presented in a green and blue color, so we further analyzed the paintings dominant in a yellow hue and summarized these to three types: (1) Depict sunrise or sunset scenery (twelve paintings). (2) Depict flowers and fields (eleven paintings). (3) Depict the architecture’s appearance in a yellow or orange hue (four paintings).

### 3.3. Element Arrangement Features

**Figure 9:**
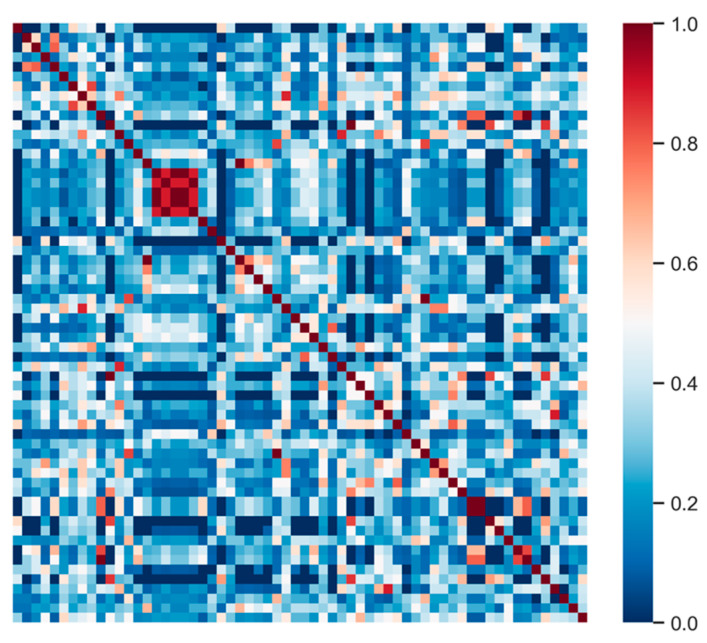
Similarity matrix of element arrangement feature of 62 paintings.

Through the Jaccard pairwise comparison, the similarity matrix of the element arrangement features was obtained and illustrated as a heat map in Figure 9.

Table 2 shows the results of element arrangement at each distance. The basic landscape categories depicted in each distance account for over 25%. In the short distance, there were Field/Grass, Forest/Tree, and Sea/Harbor, with the proportions of 48.39%, 43.55%, and 38.71%. In the medium distance, there were Sea/Harbor, Buildings, and Forest/Trees, with the proportions of 56.45%, 46.77%, and 29.03%. In the long distance, there were Mountain, Island, and Sea, with the proportions of 80.65%, 38.71%, and 37.10%. Generally, natural landscape elements were widely depicted in the coastal area of the Seto Inland Sea. In addition, green elements were always arranged in the short distance. Sea, Building, and green elements were mostly distributed in the medium distance, and Mountain was mostly arranged in the long distance.

### 3.4. Comprehensive Similarity and Classification

**Figure 10:**
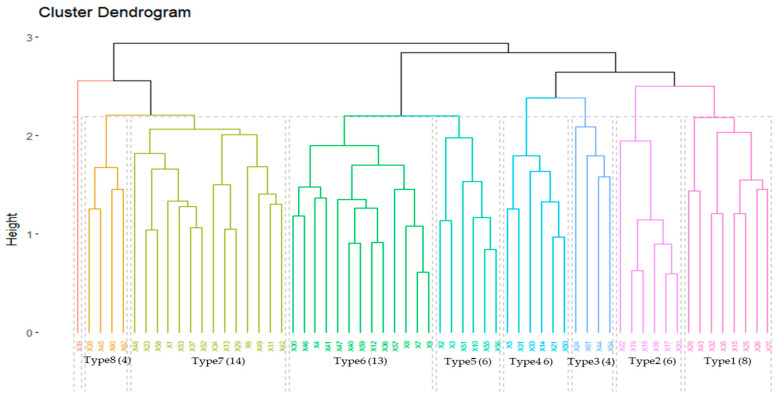
Cluster dendrogram with the complete-linkage method.

**Figure 11:**
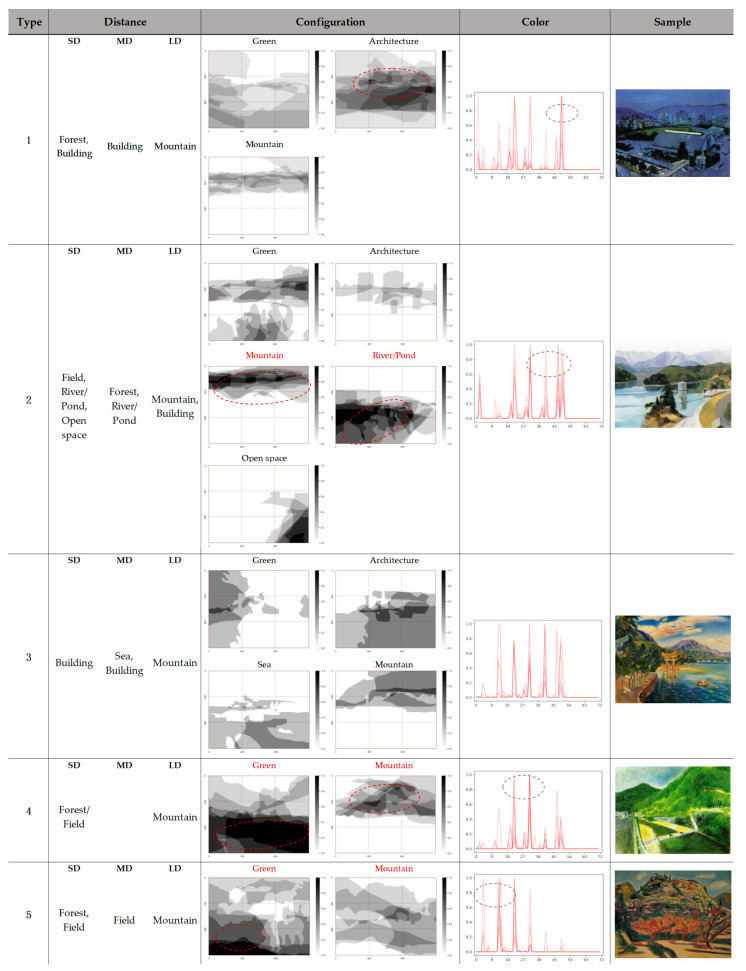

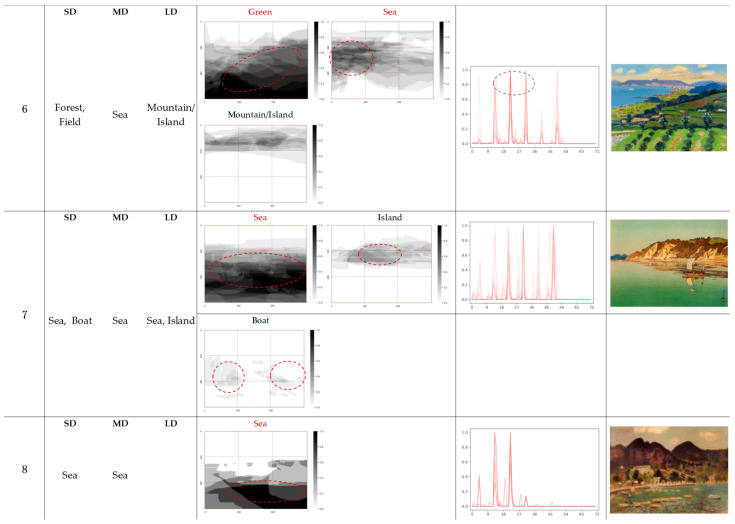
The landscape features eight types. SD: Short distance; MD: Medium distance; LD: Long distance. Red: significant elements; Black: main elements.

Based on the distance matrix and complete-linkage method, 62 paintings were classified into eight types (we skipped the type which only included one case). The dendrogram is shown in Figure 10. We extracted the landscape categories, which account for over 50% of the total in the three distances, and further analyzed the configuration feature of these landscape categories (elements). The features of each painting type of Seto Inland Sea were interpreted as follows, and we summarized the features in Figure 11.

Type 1. Architecture Landscape (Eight Paintings)

Forest and Architecture in the short distance, Architecture in the medium distance, and Mountains in the long distance were most arranged in this type of painting. Architecture dominated the entire painting and was mostly located in the middle part of the image, with Mountain in the top part and green land in the bottom part, corresponding to the categories identified at different distances. This type of painting presents rich colors, of which the blue hue showed significance. Except for medium saturation, low saturation was usually presented in paintings, accounting for about a third of the image area.

Type 2. Pond/River Landscape (Six Paintings) 

Pond scenery was highly significant in this type, with Field, Pond, and Open Space in the short distance, Forest, Pond in the medium distance, and Mountain and Architecture in the long distance. The Pond was concentrated in the bottom left-hand part of the entire image area. The mountain was depicted almost in the top part of the image instead of the sky. The color also varies in this type, especially green and blue hues, and high brightness was significant in this type of painting, accounting for almost half of the total area, which is close to medium brightness.

Type 3. Nearshore Landscape (Four Paintings)

Architecture in the short distance, Sea and Architecture in the medium distance, and Mountain in the long distance showed nearshore scenery of this type of painting. However, most of the same landscape elements were distributed in different parts of the image area in this type, suggesting a low similarity in configuration. As for the color feature, no dominant hue was presented in this type.

Type 4. Field Landscape in Green Hue (Six Paintings)

The configuration and arrangement features presented great dominance of green and Mountain. Forest and Field in the short distance and Mountain in the long distance were mostly depicted in this type. Furthermore, green and Mountain almost divided the image into two parts, with more at the bottom and less at the top. The color was significant in green hue with low and medium saturation and high and medium brightness. 

Type 5. Field Landscape in Yellow and Orange Hue (Six Paintings)

Similar to type four, green and Mountain dominated this type. However, the configuration shows a difference between these two types, of which Mountain was in the middle part in this type. In addition, the yellow and orange hues were clearly presented in type 5, which was distinct from type 4, indicating more flower landscapes or autumn and winter scenery in this type.

Type 6. Green-Seaside Landscape (Thirteen Paintings) 

Forest and Field in the short distance, Sea in the medium distance, and Mountain/Island in the long distance were widely arranged in this type. The green element covered almost half the area of the image, especially in the bottom part and the right side of the middle part, presenting a trapezoidal composition. Accordingly, the sea was concentrated on the left side of the middle part, and Mountain/Island was accompanied in the top part of the image. Yellow and green hues were mainly applied in this type.

Type 7. Island and Boat Landscape (Fourteen Paintings)

Sea and Boat in the short distance, Sea in the medium distance, and Mountain in the long distance were arranged in this type. The Sea was identified in each distance and covered almost two-thirds of the area of the image. Island was mainly distributed in the middle and top parts of the image and concentrated in the center area, suggesting that Island was distributed both on the left and right sides. The Boat was mainly distributed on the left and right sides as a point scene in the image. Rich in color and no conspicuous dominant hue was characterized in this type of painting.

Type 8. Seaside Landscape in Sunset (Sun Raise) Period (Four Paintings)

In this type of painting, the elements differed significantly both in configuration and arrangement, with only the Sea element in common, but all of them were depicted as twilight or dawn landscapes. Yellow and orange colors were clearly dominant in this type of painting, corresponding to the period in which the scenery depicts.

In general, seascapes were the most represented and varied in this area, with nearshore scenery accompanied by Building and Mountain, Island and Boat scenery, and seaside scenery depicted alongside green. These typical seascapes suggested a relationship of overall progression accompanied by partial overlap, which had similarities in some landscape elements but differed in configuration and presented a tendency from far to near in general. Moreover, besides the azure sea and blue sky, seascapes at sunset are also favored by painters. Meanwhile, field and mountain scenery also represented this area, including field, mountain, and flower scenery. In addition to the green and vibrant landscapes, the yellowish dusk and autumn landscapes were also favored by painters. Architecture landscape mainly brings attention to historical architecture or factory scenery in this area in close and medium viewing. All paintings in the pond landscape are depicted in the same scenic spot of Mano Pond. 

## 4. Discussion

In this study, we clarified the general and typical landscape features in the coastal area of the Seto Inland Sea based on landscape paintings. The results and interpretation indicated that we clarified more landscape information from landscape paintings than previous work to present the “good and characteristic landscape” better. Color features provided more information about the period and season. The configuration features of the elements indicated more element features of the aggregation degree and detailed position than in the element arrangement features. We reckon these findings can provide more comprehensive guidance and data for the subsequent landscape planning work and analysis, especially for exploring characteristic regional landscapes and developing, preserving, and evaluating tourism landscape resources in urban planning. In addition, we presented a simple and feasible method to classify landscape paintings by comminating the similarities of three aspect features. The method proposed a practical idea to combine the features or other indicators from different attribution. The indicators can be flexibly combined and substituted depending on the actual application and the research purpose.

This study only considered the features of landscape elements and colors to clarify the landscape painting. However, besides the information directly extracted from the painting, viewpoint features such as elevation features and angle features are also highly important to landscape planning work, especially in viewpoint exploration and facilities evaluation. Moreover, this study’s sample size is relatively small, only 62 cases, which resulted in the less characteristic of some typical landscapes. These are the limitations of this study and need further attention and consideration in future research. 

## 5. Conclusions

Japan passed the Landscape Law in 2004 to protect the local landscape. Since then, scholars have begun to explore local landscape characteristics through local resources. Among them, landscape painting provides a more comprehensive and objective representation of excellent and distinctive local scenery, so comprehensive research on landscape painting is fundamental and necessary for the subsequent landscape planning work. Landscape painting includes both planar information and spatial information. However, there has been little previous work on landscape painting from both three-dimensional and planar perspectives; the landscape features of landscape paintings still require to be comprehensively clarified. Seto Inland Sea region greatly influenced the development of modern–contemporary painting in Japan. Many painters were born here and left numerous realistic landscape paintings. 

Therefore, this paper, taking the Seto Inland Sea as a case study, aims to comprehensively clarify the landscape features of paintings and provide a valuable index of “good and characteristic landscapes” in this area. We selected three aspects of features highly relevant to the landscape to comprehensively analyze the landscape features of paintings; they were: (1) the element configuration, (2) the color as planar information, and (3) the element arrangement as spatial information. To further clarify the typical features of landscape paintings, we presented a feasible classification method by comminating the similarities of three aspect features. In this paper, firstly, we clarified the three general features and calculated the similarity matrices between paintings of these three features based on the respective similarity algorithms. Secondly, adding these three similarity matrices together obtained a comprehensive similarity matrix. Then, we classified these 62 paintings based on the comprehensive similarity (dissimilarity) matrix through Hierarchical Clustering Analysis.

The main contributions and findings are shown as follows:

- We comprehensively clarified the general and typical landscape features in the coastal area of the Seto Inland Sea, and more landscape information was extracted to better present the features of a “good and characteristic landscape”, which can provide more comprehensive guidance and data for the subsequent landscape planning work and analysis in this area;
- We presented a simple and feasible method to classify landscape paintings by comminating the similarities of three aspect features. The method proposed a practical idea that can flexibly combine the features or other indicators from different attribution depending on the actual application and the research purpose;
- Sky, Green, and Sea were the most essential elements of the painting. In addition, the Sky element was almost always depicted at the top part of the image, and Green and Sea elements were less depicted and were more evenly distributed in the image, of which the Sea element was more inclined to be depicted in the middle and bottom parts of the painting;
- Yellow (orange), green and blue were commonly used in target paintings. In addition, paintings were usually painted in medium and low saturation, as well as medium and high brightness. The yellow and green with medium saturation and brightness were mostly presented in landscape paintings in the coastal area of the Seto Inland Sea.
- The 62 paintings were classified into eight types; they were: (1) Architecture landscape (eight paintings), (2) Pond/River landscape (six paintings), (3) Nearshore landscape (four paintings), (4) Field landscape in green hue (six paintings), (5) Field landscape in yellow and orange hue (six paintings), (6) Green Seaside landscape (thirteen paintings), (7) Island and boat landscape (fourteen paintings), and (8) Seaside landscape in sunset (sun raise) period (fourteen paintings).

For the limitations of this study, we only considered the features of landscape elements and colors to clarify landscape paintings. Viewpoint features are also vital to landscape planning work. Moreover, this study’s sample size is relatively small, only 62 cases, which resulted in the less characteristic of some typical landscapes. Therefore, in future research, more features and samples would be analyzed, such as location feature and elevation feature of viewpoint, to deeply clarify the landscape feature index from the perspective of landscape painting. Furthermore, the priority and the utilization method of each feature in landscape planning will be further discussed in future research. 

## Figures and Tables

**Table 1:** The specific quantization scheme and function.

Attribution	Number	Range	Presentation
H	0	(316, 20]	Red, Black, White
	1	(20, 40]	Orange
	2	(40, 75]	Yellow
	3	(75, 155]	Green
	4	(155, 190]	Cyan
	5	(190, 270]	Blue
	6	(270, 295]	Purple
	7	(295, 316]	Red
S	0	[0, 0.2)	Low
	1	[0.2, 0.7)	Medium
	2	[0.7, 1]	High
V	0	[0, 0.2)	Low
	1	[0.2, 0.7)	Medium
	2	[0.7, 1]	High
G = 9H + 3S + V (0~71)			

**Table 2:** Element arrangement of 62 landscape paintings.

Distance	Landscape Category	Case Number	Proportion
Short distance	Forest/Tree	27	43.55%
	Field/Grass	30	48.39%
	River/Pond	8	12.90%
	Sea/Harbor	24	38.71%
	Boat	14	22.58%
	Building	14	22.58%
	Road/Open space	11	17.74%
Medium distance	Mountain	10	16.13%
	Forest/Tree	18	29.03%
	Field/Grass	12	19.35%
	River/Pond	7	11.29%
	Sea/Harbor	35	56.45%
	Island	11	17.74%
	Boat	13	20.97%
	Building	29	46.77%
	Bridge	8	12.90%
Long distance	Mountain	50	80.65%
	Sea	23	37.10%
	Island	24	38.71%

## Data Availability

Not applicable.

## Appendix A

**Table A1:** The Basic information of 62 paintings.

No.	Name	Author	Published Year	From
1	Naruto	Gentaro KOITO	1952	Tokushima Modern Art Museum. https://art.bunmori.tokushima.jp/srch/srch_art_sakka.php/ (accessed on 12 March 2018)
2	Sunrise of Mt. Goken	Takeji FUJISHIMA	1932	Bridgestone Museum of Art fiftieth anniversary celebration Fujishima Takeji [27]
3	Sunrise of Mt Goken	Takeji FUJISHIMA	1932	Ehime Prefectural Museum Collection 2010 [28]
4	Sunset of Uwa Sea	Yonezo SHIBATA	1977	Shibata Yonezo Art Album [29]
5	Kotohiragu	Tatsushiro TAKABATAKE	1953	Tokushima Modern Art Museum https://art.bunmori.tokushima.jp/srch/srch_art_sakka.php/ (accessed on 12 March 2018)
6	Near Nakagi Island	Hitone NOMA	1967	Ehime Prefectural Museum Collection 2010 [28]
7	Wilderness	Wasaku KOBAYASHI	1959	Wasaku Kobayashi Art Book [30]
8	Sadamisama	Wasaku KOBAYASHI	1959	Wasaku Kobayashi Art Book [30]
9	Seaside	Wasaku KOBAYASHI	1959	Tenchi Hourei Wasaku Kobayashi Art Book [31]
10	Spring of Shodo Island	Takeji FUJISHIMA	1936	Bridgestone Museum of Art fiftieth anniversary celebration Fujishima Takeji [27]
11	Returning Sail from Tesuki Island	Kunishiro MITSUTANI	1932	Tokushima Modern Art Museum https://art.bunmori.tokushima.jp/srch/srch_art_sakka.php/ (accessed on 12 March 2018)
12	View of Mt. Shima	Hitone NOMA	unknown	Tokushima Modern Art Museum https://art.bunmori.tokushima.jp/srch/srch_art_sakka.php/ (accessed on 12 March 2018)
13	Sakate Port	Kakutaro KASHIWABARA	unknown	The Kagawa Museum art collection catalog [32]
14	Kuribayashi Park	Toshio NAKANISHI	1936	Tokushima Modern Art Museum https://art.bunmori.tokushima.jp/srch/srch_art_sakka.php/ (accessed on 12 March 2018)
15	Takamatsu City	Mitsuyuki NAKAMURA	1979	The Kagawa Museum art collection catalog [32]
16	Manno Pond	Yoshisumi KONISHI	1990	The Kagawa Museum art collection catalog [32]
17	Manno Pond	Yoshisumi KONISHI	1995	The Kagawa Museum art collection catalog [32]
18	Manno Pond	Yoshisumi KONISHI	1998	The Kagawa Museum art collection catalog [32]
19	Manno Pond	Yoshisumi KONISHI	1990	The Kagawa Museum art collection catalog [32]
20	Manno Pond	Yoshisumi KONISHI	2001	The Kagawa Museum art collection catalog [32]
21	Mount Iino	Norimasa AMAMIYA	1981	The Kagawa Museum art collection catalog [32]
22	Sakaide Harbor Scenery	Chutsu IMANISHI	unknown	Tokushima Modern Art Museum https://art.bunmori.tokushima.jp/srch/srch_art_sakka.php/ (accessed on 12 March 2018)
23	From Megi Island to Look Over Nao Island	Shintaro YAMASHITA	1934	Tokushima Modern Art Museum https://art.bunmori.tokushima.jp/srch/srch_art_sakka.php/ (accessed on 12 March 2018)
24	Ya Island	Ken KAGEYAMA	unknown	The Kagawa Museum art collection catalog [32]
25	Zentsuji Temple	Masajiro KAWAZOE	1980	The Kagawa Museum art collection catalog [32]
26	Copper Refinery in Nao Island	Shigeyoshi KAWAHITO	1980	The Kagawa Museum art collection catalog [32]
27	Oil Storage in Shodo Island	Hirokatsu KINOSHITA	1980	The Kagawa Museum art collection catalog [32]
28	Otomon of Marugame Castle	Shoroku KOBAYASHI	1982	The Kagawa Museum art collection catalog [32]
29	Mt. Goken	Asaji SAITO	1979	The Kagawa Museum art collection catalog [32]
30	Seto Island in Summer	Heizo KANAYAMA	1916	The National Museum of Modern Art https://www.momat.go.jp/collection/ (accessed on 9 April 2018)
31	Shioe Hot Spring Village	Sadao TOMIIE	1981	The Kagawa Museum art collection catalog [32]
32	Inokuma Residence	Eisuke NAKAMURA	1979	The Kagawa Museum art collection catalog [32]
33	Inogono Pass in Autumn	Yoshimori FUNAOKA	1980	The Kagawa Museum art collection catalog [32]
34	Tsushima	Ra MUKAI	unknown	The Kagawa Museum art collection catalog [32]
35	Kanamaluza	Takayoshi OKADA	unknown	The Kagawa Museum art collection catalog [32]
36	Scenery of Seto Island Sea in Early Summer	Kakutaro KASHIWABARA	1974	The Kagawa Museum art collection catalog [32]
37	View from Ya Island	Takeji FUJISHIMA	1932	Tokushima Modern Art Museum https://art.bunmori.tokushima.jp/srch/srch_art_sakka.php/ (accessed on 12 March 2018)
38	View from Ya Island	Takeji FUJISHIMA	1932	Tokushima Modern Art Museum https://art.bunmori.tokushima.jp/srch/srch_art_sakka.php/ (accessed on 12 March 2018)
39	Summer	Torajiro KOJIMA	1913	Landscape paintings of Chugoku region--From Setouchi to Sanin area [33]
40	Onomichi Scenery	Shimizu TOSHI	1933	Landscape paintings of Chugoku region-From Setouchi to Sanin area [33]
41	Kamikiri Island Scenery	Minami KUNZO	1949	Landscape paintings of Chugoku region-From Setouchi to Sanin area [33]
42	Aki Seashore	Minami KUNZO	unknown	Landscape paintings of Chugoku region-From Setouchi to Sanin area [33]
43	Structures Along the River	Ishii HAKUTEI	1927	Landscape paintings of Chugoku region-From Setouchi to Sanin area [33]
44	Itsuku Island	Tokusaburo MASAMUNE	unknown	Landscape paintings of Chugoku region-From Setouchi to Sanin area [33]
45	Itsuku Island	Shintaro SUZUKI	1953	Landscape paintings of Chugoku region-From Setouchi to Sanin area [33]
46	Mount Taika	Ebihara KINOSUKE	1953	Landscape paintings of Chugoku region-From Setouchi to Sanin area [33]
47	Olives and Sea	Toku SATAKE	1965	Landscape paintings of Chugoku region-From Setouchi to Sanin area [33]
48	Lighthouse	Shiyohei MATSUDA	1959	Landscape paintings of Chugoku region-From Setouchi to Sanin area [33]
49	Dannoura	Ishikawa TORAJI	1963	Landscape paintings of Chugoku region-From Setouchi to Sanin area [33]
50	Mt. Naki	Atsushiro KOBAYAKAWA	1951	Landscape paintings of Chugoku region-From Setouchi to Sanin area [33]
51	Mt. Akikame	Kunitaro SUDA	unknown	Landscape paintings of Chugoku region-From Setouchi to Sanin area [33]
52	Kaminoa Island	Hiroshi YOSHIDA	1930	Landscape paintings of Chugoku region-From Setouchi to Sanin area [33]
53	Shirashi Island	Hiroshi YOSHIDA	1922	Landscape paintings of Chugoku region-From Setouchi to Sanin area [33]
54	Tomo Harbor	Hiroshi YOSHIDA	1930	Landscape paintings of Chugoku region-From Setouchi to Sanin area [33]
55	Plum	Wasaku KOBAYASHI	1935	Wasaku Kobayashi Art Book [30]
56	Island of Spring	Wasaku KOBAYASHI	unknown	Wasaku Kobayashi Art Book [30]
57	The Sea in Springtime	Wasaku KOBAYASHI	1974	Wasaku Kobayashi Art Book [30]
58	Sensui Island	Torajiro KOJIMA	1917	Landscape paintings of Chugoku region-From Setouchi to Sanin area [33]
59	Landscape of Island	Wasaku KOBAYASHI	unknown	Tenchi Hourei Wasaku Kobayashi Art Book [31]
60	Tomotu Fish Market Times	Kunishiro MITSUTANI	unknown	Landscape paintings of Chugoku region-From Setouchi to Sanin area [33]
61	Cloudy Day of Harbor	Yoson IKEDA	1914	Landscape paintings of Chugoku region-From Setouchi to Sanin area [33]
62	Moring of Sensui Island	Umehara RYUZABURO	1932	Landscape paintings of Chugoku region-From Setouchi to Sanin area [33]
